# *Scolopostethus affinis* (Schilling) (Hemiptera, Heteroptera, Rhyparochromidae, Drymini): a new alien established in North America

**DOI:** 10.3897/zookeys.889.35805

**Published:** 2019-11-14

**Authors:** David J. Larson, Geoffrey G.E. Scudder

**Affiliations:** 1 Box 56, Maple Creek, Saskatchewan, S0N1N0, Canada Unaffiliated Maple Creek Canada; 2 Department of Zoology and Centre for Biodiversity Research, and University of British Columbia, 6270 University Boulevard, Vancouver, BC, V6T 1Z4, Canada University of British Columbia Vancouver Canada

**Keywords:** Canada, Hemiptera, Rhyparochromidae, *Scolopostethus affinis*

## Abstract

*Scolopostethus affinis*, a species native to the Palearctic region, is reported from two localities in Montreal, Quebec. The species appears established and breeding in Quebec and is a new alien species in North America. A description of *S. affinis* is given, with illustrations, and details of the life cycle and diagnostic characters.

## Introduction

The genus *Scolopostethus* Fieber (Rhyparochromidae: Rhyparochrominae: Drymini) comprises a group of 34 relatively small species that occur within the Holarctic and northern Ethiopian biogeographic regions (Slater 1964, Dellapé and Henry 2019). One species, *S. thomsoni* Reuter, is Holarctic occurring across Northern North America, four are Nearctic (Henry and Froeschner 1988) and 29 are Palearctic.

*Scolopostethus affinis* (Schilling) is an Old-World species, a Euro-Siberian element in the fauna, known from all of Europe, including Russia, with recorded occurrence in Asia (Péricart 1998). In the British Isles, it is quite common, and occurs on a wide variety of soils where it is often associated with nettles (*Urtica dioica* L.) (Southwood and Leston 1959). It is usually brachypterous, but macropters are sometimes present (Southwood and Leston 1959).

There is an extensive literature on *S. affinis*, as documented by Slater (1964) and Dellapé and Henry (2019). Slater (1964) lists the references that give illustrations. In the recent literature, a colour illustration is included in Southwood and Leston (1959), and Péricart (1998) provides illustrations relevant for accurate identification. The latter two authors provide keys to European species. Larson and Scudder (2018) describe and illustrate the two species then recognised on the prairies and in eastern Canada and discuss previously reported species.

Eyles (1963a) described the life cycle of *S. affinis* in England and concluded that it has a single generation a year, but Southwood and Leston (1959) state that it is either single-brooded, with a long oviposition season, or double-brooded. Eyles (1963b) describes the egg and the five immature stages, although these immature stages cannot be separated from those of *S. thomsoni* and have a similarly timed life cycle. However, Eyles and Blackith (1965) found that *S. affinis* did not successfully hybridise with other species of *Scolopostethus*.

Eyles (1964) showed that seeds are the principal item of food in the diet of *S. affinis.* When larvae were reared on different seeds, seeds of the stinging nettle (*Urtica dioica* L.) produced the most productive growth curve. Eyles (1963a) found that *S. affinis* in England overwinters in nettle litter or in leaf litter near nettles, and was most readily collected in nettles. Eyles (1964) showed that although *S. affinis* survived to the third instar on a diet of beetle larvae, it scarcely showed any growth.

## Materials and methods

Quebec specimens have been compared with material collected in England and Germany. Measurements were obtained from this European material, but total length of Quebec specimens is also given.

Quebec specimens are deposited in the following collections:

**CNC**Canadian National Collection, Agriculture and Agri-Food Canada, Ottawa;

**DJL** D.J. Larson collection;

**GGES** G.G.E. Scudder collection.

All European specimens studied are in the latter collection.

## Taxonomy

### Scolopostethus affinis

Taxon classificationAnimalia

(Schilling, 1829)

413312D2-ACEA-589D-9A08-0FB04A71DC15

Fig. 1

Pachymerus affinis Schilling, 1829, Beitr. Ent. Schles. Fn. 1: 80.
Scolopostethus affinis : Stål 1862, Ofv. Vet. Akad. Forh. 10: 219 (current combination).
Scolopostethus adjunctus Douglas & Scott, 1865, Brit. Hem. Het.: 183 (synonym).
Scolopostethus affinis : Slater 1964, Cat. Lygaeidae World 2: 954 (bibliography).
Scolopostethus affinis : Péricart 1998, Fauna de France 84B: 301 (description).
Scolopostethus affinis : Dellapé & Henry, Lygaeoidea Species File, Ver. 5.0/5.0 (2019) (bibliography, distribution).

#### Description.

***Colour.*** Head fuscous; antennae with first and second segments completely pale ferruginous, third antennal segment basally pale ferruginous, with rest of third segment and whole of fourth segments dark brown; rostrum with first segment fuscous, second and third segments ochraceous, and fourth segment ferruginous. Pronotum with anterior lobe of disc fuscous, posterior lobe ferruginous with humeral angles and patch on each side of midline dark brown; anterior margin of pronotum dark ferruginous; lateral carina dusky anteriorly, but pale ochraceous at level of transverse impression, and more or less fuscous posteriorly. Scutellum fuscous. Hemelytra flavo-ochraceous, with posterior third of corium fuscous and streaks of fuscous extending basally along R+M, along subcostal area and along second row of punctures adjacent to scutellum; corium with a distinct quadrate fuscous spot about middle and almost or actually contacting costal margin; clavus with apical fuscous streak between middle rows of punctures; membrane pale with veins fuscous. Legs flavescent, with fore femora medially dark brown. Venter fuscous with anterior margin of prosternum, posterior margin of proplura and metapleura, and coxal covers (= acetabula) flavo-ferruginous.

***Structure.*** Head punctate; rostrum reaching to or almost to middle coxae, with first segment reaching to or almost to anterior margin of prosternum. Pronotum punctate, especially near anterior margin; pronotum of brachypterous forms rather quadrate and slightly concave at level of transverse impression: pronotum in macropters distinctly wider than long and distinctly concave laterally; anterior lobe of disc longer than posterior lobes, in most cases twice as long. Hemelytra in brachypters reaching middle of tergum VI, in macropters reaching virtually to end of abdomen. Clavus with three rows of regular punctures, plus a few odd punctures in apical half between the two rows nearest scutellum. Fore femora incrassate, with rows of small spines both antero- and postero-ventrally, the antero-ventral row extending along most of femora with small spines both distal and proximal to the larger spine, found on the apical third of femora. Fore tibia of male distinctly curved, with apex expanded and with small spines. Mesosternum of male with a pair of curved processes, replaced in female by short tubercles, best seen in side view.

Measurement of British specimens (all measurements in mm):

**Male brachypter** (*N* = 10). Head width: 0.76 (0.70–0.82).

Antennal measurements: 0.38 (0.37–0.43): 0.64 (0.62–0.70): 0.59 (0.56–0.60): 0.67 (0.13–0.70).

Pronotal width: 1.16 (1.07–1.23) pronotal length 0.95 (0.90–1.07).

Total length: 3.79 (3.60–3.80).

**Male macropter**. Head width: 0.79 (0.75–0.83).

Antennal measurements: 0.38 (0.37–0.40): 0.61 (0.60–0.63): 0.57: 0.65 (0.63–0.67).

Pronotal width: 1.14 (1.00–1.25), pronotal length 0.82 (0.77–0.87).

Total length: 3.80 (3.50–4.04).

**Female macropter** (*N* not recorded). Head with: 0.82 (0.80–0.83).

Antennal measurements: 0.37 (0.36–0.40): 0.62 (0.60–0.63): 0.58 (0.57–0.62): 0.67 (0.65–0.70).

Pronotal width: 1.32 (1.28–1.38), pronotal length 0.91 (0.90–0.92).

Total length: 4.13 (4.00–4.25).

**Measurements of Quebec specimens**

Total length: male (*N* = 10) 3.40–3.77; female (*N* = 10) 3.70–4.14.

Percent brachypterous: male 85.7 % (*N* = 21); female 63.6% (*N* = 11).

#### Diagnosis and remarks.

**Figure 1.**
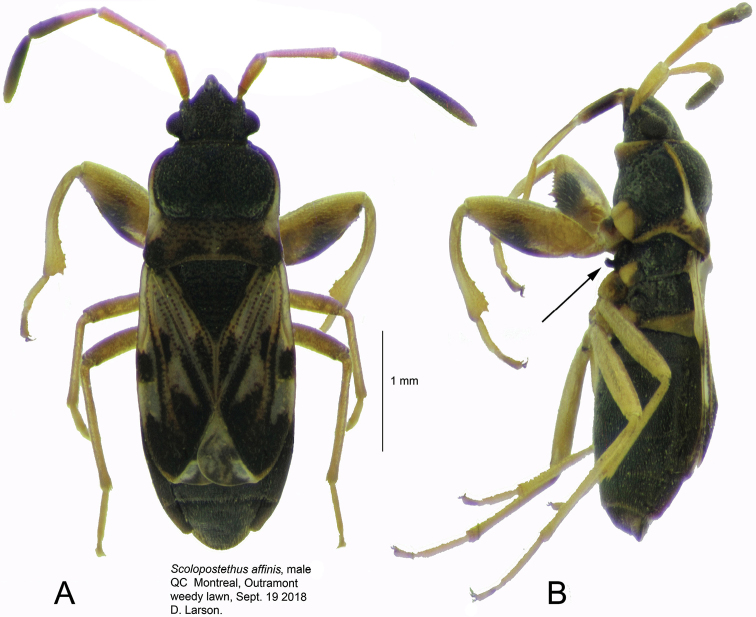
*Scolopostethus affinis*, male brachypter. **A** dorsal aspect **B** lateral aspect, arrow indicates mesosternal process.

The species is easily recognised and distinguished from Nearctic species of *Scolopostethus* by the pair of curved processes on the mesosternum of males (Fig. 1B), and the short tubercles on the mesosternum of females in front of the middle coxae, which are best seen in the side view of thorax. The antennae are distinct (Fig. 1) in that the second segment lacks a fuscous apex and is unicolourous whereas in *S. thomsoni* Reuter, the second antennal segment has the apex clearly fuscous.

The fore femora of *S. affinis*, like *S. thomsoni*, have a row of short spines both antero- and postero-ventrally, with the antero-ventral row with short spines both proximal and distal to the larger spine.

#### Material examined.

Quebec material examined is as follows:

17♂ 8♀, QUEBEC, Montreal, Outremont, weedy lawn, 29.ix.2018 (D. Larson) [CNC, DJL, GGES].

5♂ 1♀, QUEBEC, Montreal, Olympic Park, 24.ix.2018 (D. Larson) [DJL, GGES].

## Discussion

This species was collected in Outremont, Quebec, in a ruderal area around a newly constructed building, with many weeds, including dandelion (Asteraceae: *Taraxacum* sp.), knapweed (Asteraceae: *Centaurea* sp.), nettles (Urticaceae: *Urtica dioica* L.), burdock (Asteraceae: *Arctium* sp.), and lamb’s quarters (Amaranthaceae: *Chenopodium album* L.). Although late-instar nymphs were abundant, proving the species was present as a reproducing colony, no specimens were retained.

The true bug fauna of Quebec is well known. If this apparently synanthropic species had been present in Quebec for any length of time, it is highly probable it would have been discovered before now. Thus, *S. affinis* is likely a recent introduction into the fauna. As the species has a wide range in the Palearctic, indicating it can survive in diverse environments, and is clearly adapted to some types of human-modified environments, it is likely to persist and spread in North America. The North American population contains both brachypters and macropters so rapid dispersal by flight is a possibility but also synanthropic species have the advantage of human assisted dispersal so that rapid spread is possible. Fortunately, this species is not known to have any deleterious effects on crops and its propensity of feeding on seeds of plants we regard as weeds may make it a welcome addition to our environments. It is not known how it might interact with North American species although it does coexist with a diverse fauna of Palearctic *Scolopostethus*, which includes the Holarctic *S. thomsoni*, so it may occupy an uncontested niche.

## Supplementary Material

XML Treatment for Scolopostethus affinis
